# The interplay between the urban development of Rome (Italy) and the Tiber River floods: A review of two millennia of socio-hydrological history

**DOI:** 10.1007/s13280-024-02078-5

**Published:** 2024-10-05

**Authors:** Elena Ridolfi, Mara Lucantonio, Giuliano Di Baldassarre, Benedetta Moccia, Francesco Napolitano, Fabio Russo

**Affiliations:** 1 Dipartimento di Ingegneria Civile, Edile e Ambientale, Università Degli Studi di Roma La Sapienza, 00184 Rome, Italy; 2 Department of Earth Sciences, Uppsala University, Uppsala, Sweden; 3 Centre of Natural Hazards and Disaster Science, Uppsala, Sweden

**Keywords:** Adaptation, Flood risk, Levee system, Flood risk mitigation, Socio-hydrology, Long-term interaction

## Abstract

The urban development of Rome (Italy) has been intertwined with the dynamics of the Tiber River since its foundation. In this review paper, we analyse more than 2500 years of flood history and urban development to untangle the dynamics of flood risk and assess the resulting socio-hydrological phenomena. Until the 1800s, urban dwellers living in the riparian areas of the Tiber River were accustomed to frequent flooding. From the 1900s, the construction of flood walls reshaped the co-evolution of hydrological, economic, political, technological, and social processes. As a result, while the probability of flooding is currently very low, its potential adverse consequences would be catastrophic. From the analysis of the long-term feedback between urban development of Rome and flood events from ancient times to present days, it emerges the crucial need for an effective flood risk mitigation strategy that combines structural and non-structural measures. In particular, heightened flood risk awareness and preparedness to cope with rare but potentially devastating events is key to alleviate flood risk.

## Introduction

Throughout history, the development of the city of Rome has been interconnected with the Tiber River and its flood events (Aldrete 2007). The myth of Rome’s foundation, for example, tells the story of the two newborn twins, Romulus and Remus, abandoned on the riparian areas of the Tiber River during a flood event and rescued by a she-wolf that raised them. According to the myth, the basket with the twins was floating along the Tiber River and came to a stop just downstream from the Tiber Island. This specific point was crucial to control the trade in the area, making it a key location for the foundation of what would later become the Roman empire. Later, Romulus and Remus founded the city of Rome and Romulus became its first king (Plut. Rom. 2,6).

The relationship between Rome and the Tiber was so intertwined and imbued with sacred significance that in the Aeneid, Virgil, one of Rome's greatest poets, describes a personified Tiber appearing to Aeneas, the ancestor of the twins, to predict the majesty of Rome (Aen. 8.31–85). From the narratives, it is evident that Romans identify themself with the Tiber River.

The city of Rome is emblematic of the role historically played by rivers in the development of human settlements (Bird et al. 2016; Nardi et al. 2019). In modern times, most large cities lay close to major rivers as floodplain areas offer favourable conditions for socio-economic development, cultural organization, trade, and transportation and access to water resources (Hu et al. 2018).

Since settling on floodplain areas may have disastrous consequences when flooding occurs, societies strive to minimize flood risk, resulting from the product of various factors, including the hazard (e.g. floodwater levels and their probabilities), exposure (e.g. assets in the flooding area), and vulnerability (e.g. susceptibility of the assets to flooding). To mitigate flood risk, societies attempt to reduce one or more of these factors. However, the literature has shown that a change in one of these factors may affect the others due to feedback mechanisms in the human-water system and may result in unintended consequences (Barendrecht et al. 2017). Flood mitigation strategies involve implementing a combination of structural and non-structural measures. Structural measures, such as levees or flood-control reservoirs, aim to reduce the frequency of flooding. In doing so, they alter flood risk perception (complacency) and enable intense urbanization in floodplain areas. This phenomenon, known in literature as the “levee effect” (White 1945), can lead to catastrophic losses when the levees eventually fail (Lawrence et al. 2020). In some instances, the false sense of safety may encourage people to delegate flood prevention actions to the authorities (Sturloni et al. 2019). Non-structural measures aim to reduce the potential damage of flooding (rather than its frequency) by raising awareness and preparedness among the population (Maidl and Buchecker 2015), limiting urban development in the floodplain or resettling (Ridolfi et al. 2021). Resettlement outside of risky areas proved to be difficult for populations heavily reliant on structural protection measures (Hino et al. 2017). In these cases, the response to flood events often consists of reinforcing these structures (Mård et al. 2018).

Flood risk management is effective only if the three components of risk (hazard, vulnerability, and exposure) are reduced overall (Kreibich et al. 2022). Thus, it is crucial to consider the feedback within coupled human-flood systems for a reliable flood risk assessment and for effective risk reduction. This approach goes beyond the traditional method where either the effect of floods on society is estimated, for example, through damage assessment (Merz et al. 2010), or conversely, the effect of human interventions on floods is analysed, for example, assessing the impact of land-use change on floods (Raaijmakers et al. 2008). “While human societies shape hydrological extremes, hydrological extremes, in turn, shape human societies” (Di Baldassarre et al. 2017a). Examples of the long-term interactions between human and river systems have been observed in several cities around the world. For instance, Vienna has adapted and responded to flood-related challenges for centuries. As the city expanded, it extended into lower flood-prone areas, leading to the implementation of structural interventions, including eighteenth-century embankments and later canals for improved transportation and water-level management (Barendrecht et al. 2017). In Czech Republic, the Vltava River has historically influenced settlement patterns. Settlements were established farther and closer to the river, driven by the occurrence (or absence) of major flood events. Prolonged periods of “calm” faded the memory of locals who relocated closer to the river, as their risk awareness diminished over time (Fanta et al. 2019). Later, this pattern was disrupted by the construction of riverbanks, providing a sense of security, and enabling people to reside closer to the river. For over two thousand years, the Seine River and the city of Paris have shared an enduring relationship (Beaudouin 1993). This connection has encompassed a diverse interplay of cultural elements that have evolved over time, including the river's use and taming through flood control measures to meet the needs of locals (e.g. urban expansion, industry, transport, energy, and flood control; Lestel et al. 2023).

In Rome, the erection of flood walls reconfigured the intertwined dynamics of hydrology, economics, politics, technology, and society. Although the probability of a flood event is extremely low, the potential catastrophic consequences of such flooding cannot be underestimated. Understanding the mutual interaction between society and hydrological processes over time is key to planning appropriate flood risk mitigation measures in the long term. In this framework, Rome is a rare case study with reliable and long-time series of hydrological and social data (Bencivenga et al. 1995; Adrete 2007) that can be exploited to understand the dynamics of flood risk and the feedback mechanism between social and hydrological processes. While many researchers have investigated this interaction either in cities, such as Vienna (Baredrecht et al. 2017), or in large areas in Europe, e.g. Czech Republic (Fanta et al. 2019), and in China (Chen et al. 2012), an extensive analysis covering more than two millennia of history of an European city since its foundation is missing.

In this paper, we build on previous work by Di Baldassarre et al. (2017b) and Ciullo et al. (2017), who analysed human–flood interactions in Rome over the past 150 years, to uncover the causal processes between human and water systems from the early ages of Rome to modern times, covering over 2,500 years of history. The novelty of this work lies in the examination of the reciprocal relationship between the Tiber River and the Roman inhabitants, placing emphasis on the historical evolution of attitudes and perceptions regarding flood risk, spanning from antiquity to modern times.

In the following, we trace the interplay between Rome and the Tiber, undertaking an integrative literature review to identify and examine the changing relationship over two millennia. We then capture the spatial development of the city over time and analyse the attitude toward flooding across centuries. Finally, we focus on contemporary flood risk management.

## Study area

**Fig. 1:**
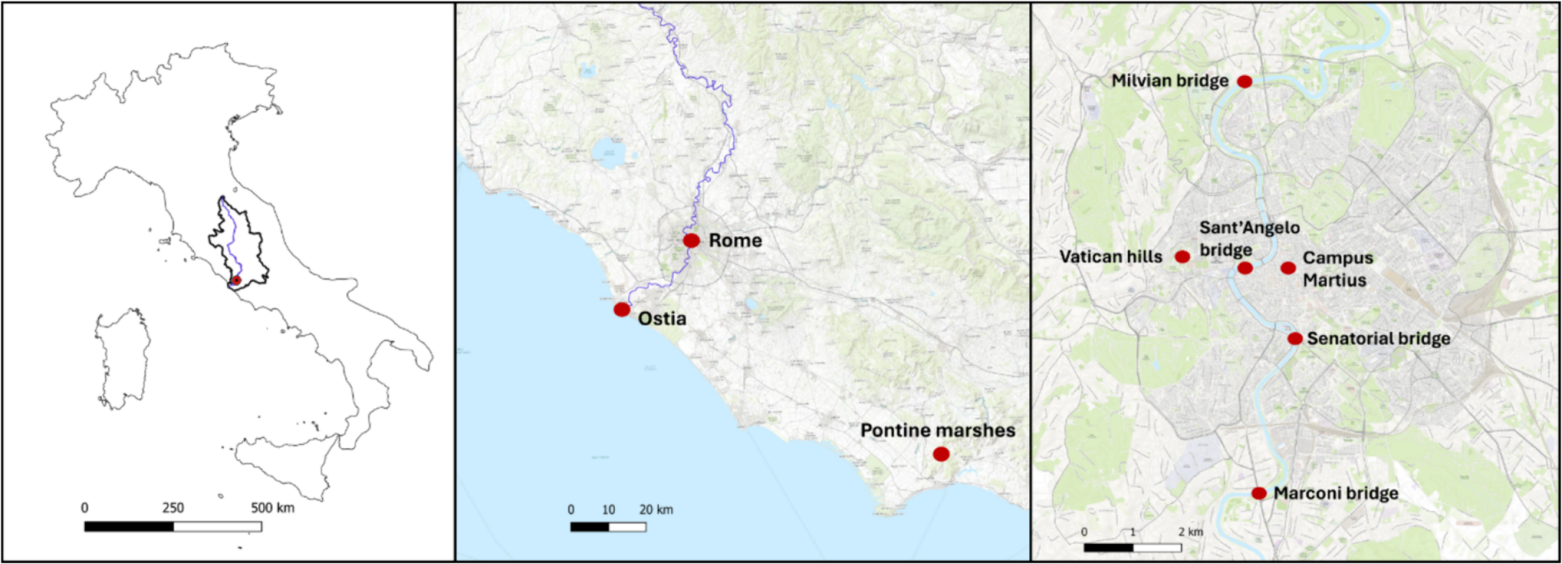
The Tiber River catchment in central Italy and Rome represented as a red circle (left panel) with two focus maps from the Italy-scale geographic location: the city of Rome, Ostia, and the Pontine marshes (middle panel) and the zoomed region of Rome (right panel)

The Tiber River is the main river of central Italy and the third longest river on the peninsula, with a basin covering an area of 17,375 km^2^. It originates from the Fumaiolo mountain at 1,268 m above sea level in Emilia Romagna, central Italy, and then flows through the territories of six regions. At the end of its course, within the Lazio region, the Tiber crosses the city of Rome and flows into the Tyrrhenian Sea via a delta with two branches, Fig. 1.

The Tiber valley separates two large geological complexes, the volcanic system of the Sabatini to the north and the Colli Albani to the south (Gisotti 2016). These geological conditions affect the relationship between water infiltration into the subsoil and surface flow. The most permeable sectors are the Apennine carbonate ridge in the eastern sector, while moderately permeable volcanic formations characterize the southwestern sector. In the northern area of the basin, there are low permeability flyschoid formations.

### History of Rome in brief: from the foundation to Rome capital of Italy

In its stretch from Rome to the mouth, the Tiber lengthens instead of deepening, causing the canal to assume a meandering configuration (Remedia 2016). The site was particularly hospitable for settlements because of its geomorphology. It was easily defensible against enemy attacks due to the presence of natural reliefs with steep slopes and flat tops, surrounded by marshy areas. The abundance of springs, small streams, and the Tiber itself, which also served as an important route for communication and transportation, as well as a source of food and drinkable water at the time, encouraged settlements.

**Fig. 2:**
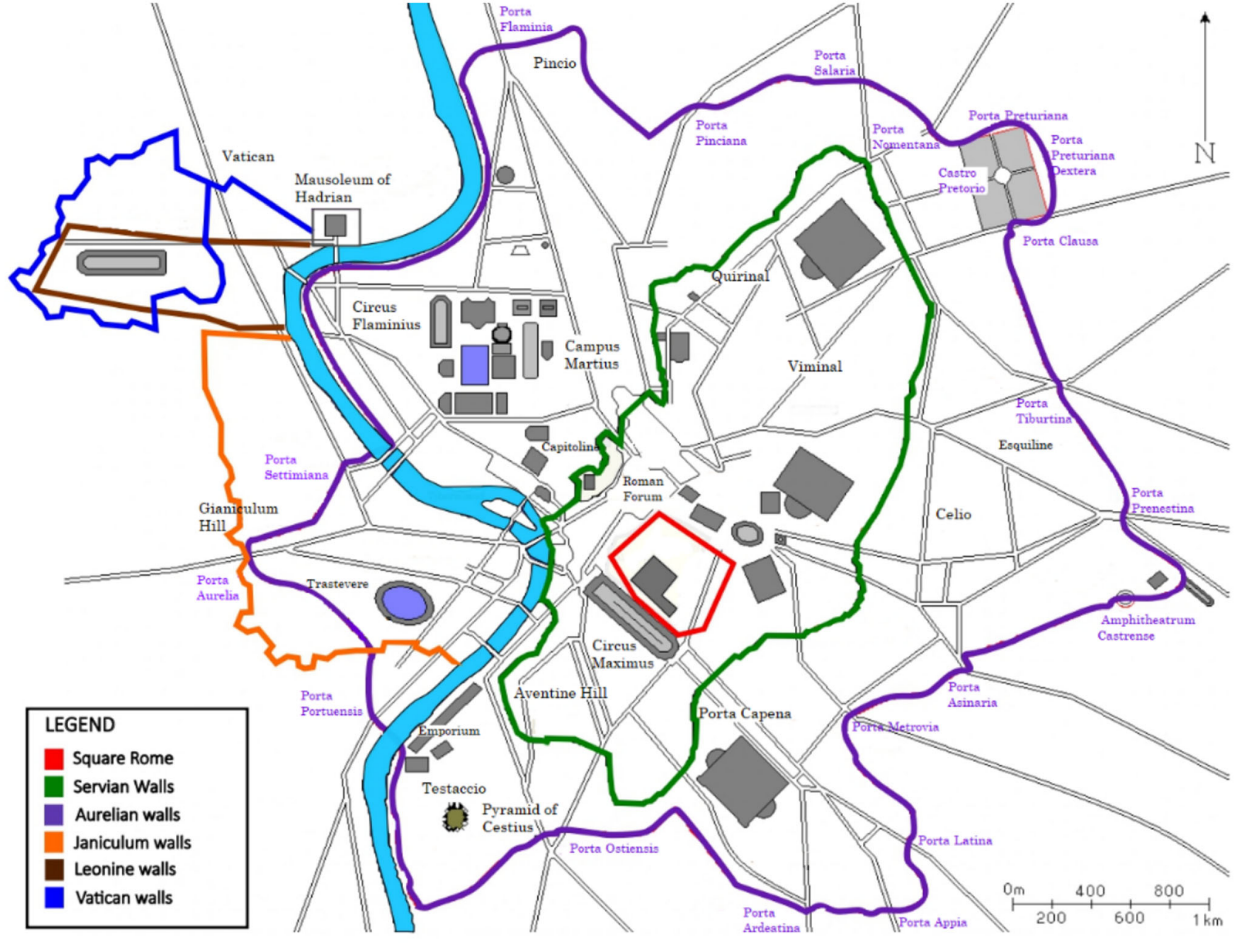
Extent of the Roman Walls over the centuries (after Canina, 2015)

The first settlement of Rome refers to an area known as Square Rome (Fig. 2 in red), as it was situated on top of Mount Palatine and had a vaguely squared shape (Tacitus, Ann., XII, 24). The city's morphology made use of the natural conformation of the hill, and therefore, no walls were needed, as the hill itself provided sufficient defence from enemies. In the sixth century BC, the city expanded and was enclosed by the Servian Walls (Fig. 2 in green). In the third century AD, barbarian invasions, migrations into Roman territory, civil wars, rebellions, and political instability led to the Imperial crisis. Feeling the urgent need for protection, Emperor Aurelian began the construction of a new city wall known as the Aurelian Walls (Dey 2011) to defend Rome from barbarian threats (Fig. 2 in purple).

Starting from 848, after the Saracenic sack of the ancient Saint Peter's Basilica, the pope erected the Leonine Walls to protect the basilica itself and the adjacent territories (Fig. 2). In the seventeenth century, the city expanded further on the right bank with the construction of the Janiculum Walls (Fig. 2 in orange).

On September 20, 1870, the Italian 'Resurgence' led to the unification of the Italian Peninsula under the Kingdom of Italy. At that time, Rome retained the urban layout designed by Pope Sixtus V in the 1500s. The urban settlement was encircled by the Aurelian Walls and had medieval characteristics. In 1871, Rome became the capital of the Italian Kingdom, marking the beginning of a new era of modernization.

### Historical floods and population

The knowledge of flood frequency is essential for addressing their impact on society, while the frequency of flood events is crucial for building up the memory of past flood events. Frequent events may raise population's awareness, as they foster the adoption of risk mitigation strategies and promote a participatory approach. Nevertheless, flood magnitude is another relevant characteristic: if the interarrival time of major floods is short, then high-magnitude events will significantly affect how inhabitants react and adapt to these occurrences (Ridolfi et al. 2020).

The impressive documentation available on the city of Rome, spanning from the earliest ages of the Roman era, allows for a unique assessment and analysis of flood frequency and magnitude in the city of Rome with the aim of understanding the dynamics of flood risk over centuries.

**Fig. 3:**
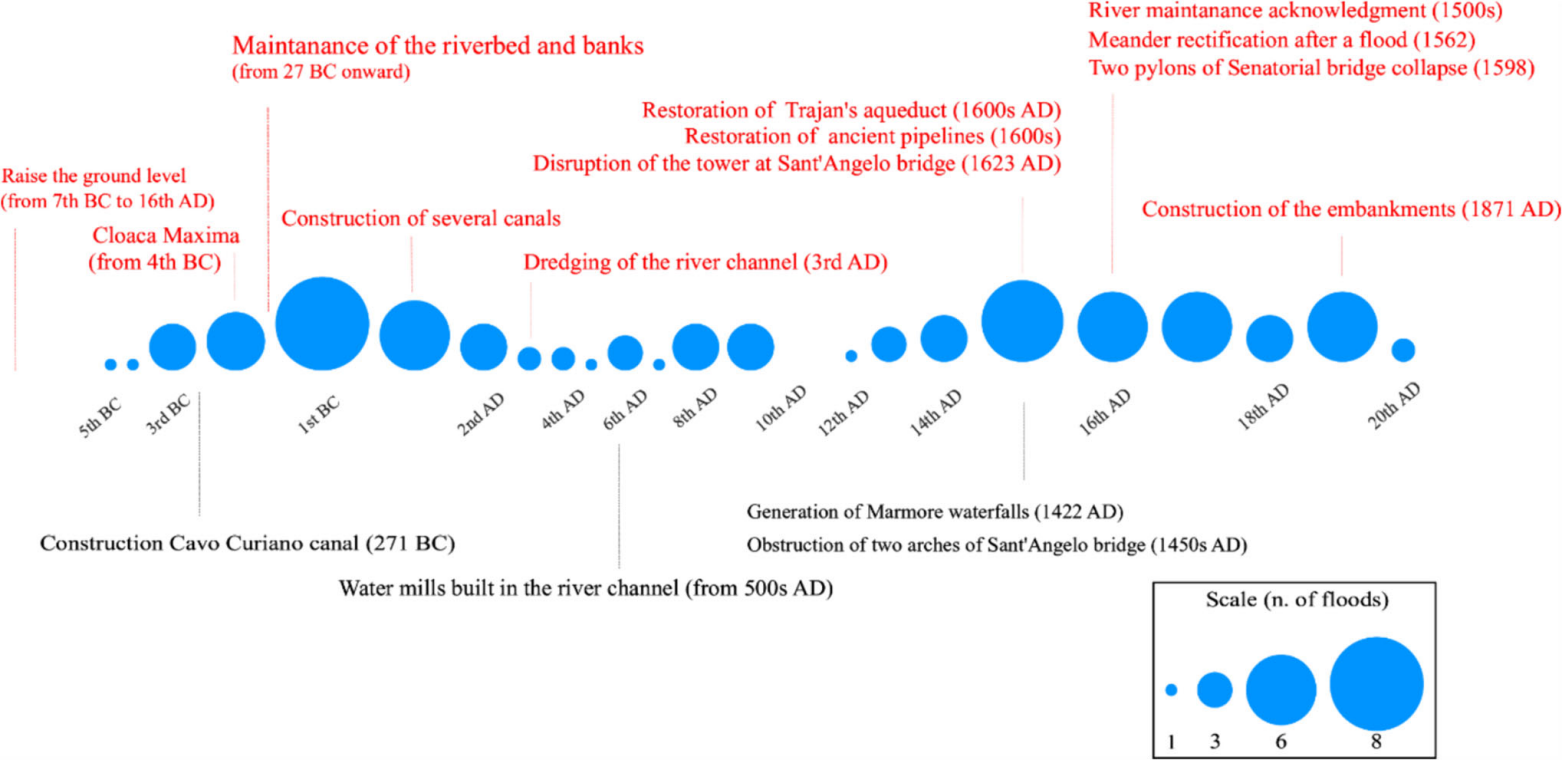
Frequency of major floods at Rome per century (circle size represents the number of floods per century) and main events that either may have caused a flooding (lower lines in black) or may have decreased the probability of flooding, upper lines in red (Aldrete 2007; Bersani and Bencivenga 2001)

Flood occurrences in Rome was previously studied by a frequentist approach, i.e. dividing a given time span by the number of attested floods (Calenda et al. 2009; Bencivenga et al. 1995). Figure 3 shows the number of flood events that occurred from the fifth century BC to the twentieth century AD. It is worth noting that some flood events may have been caused by anthropogenic changes in the river reach or the river channel, as highlighted in Fig. 3.

Many scholars agree on the low reliability of the reconstruction of the number of floods (e.g. Aldrete 2007; Bersani and Bencivenga 2001), which can be attributed to the limited number of surviving sources. In fact, the highest number of floods occurred in the first century BC, one of the most well-documented eras in terms of the amount of surviving primary sources. Primary authors did not report the water height; however, they described the effects of flooding on the streets, allowing for the reconstruction of water height. For instance, they narrated the necessity of traveling by boat, suggesting that the water height was half a meter or above (Tacitus Hist. 1.86) or that the force of the floodwater was capable of moving animals and people, indicating that the height was below half a meter (Dio 53.20.1, 53.33.5, 54.25.2, 55.22.3, 57.14.7–8). Numerical values are available only from the Middle Ages onward, when evidence was provided by the many high-water marks deployed across the city. Since 1782, the hydrometric height of the Tiber River has been measured by the first hydrometer at Ripetta river cross section, in the city centre. The combination of ancient sources with modern water-level measurements allows for the reconstruction of an impressive number of flood events. Nevertheless, an uneven distribution pattern emerges in Fig. 3 as the number of floods varies enormously across centuries.

The scarcity of surviving sources can be seen as a phenomenon connected with the population density inhabiting the city during a given historical period. This hypothesis may explain the lack of medieval data, as the population of the city indeed decreased significantly.

**Fig. 4:**
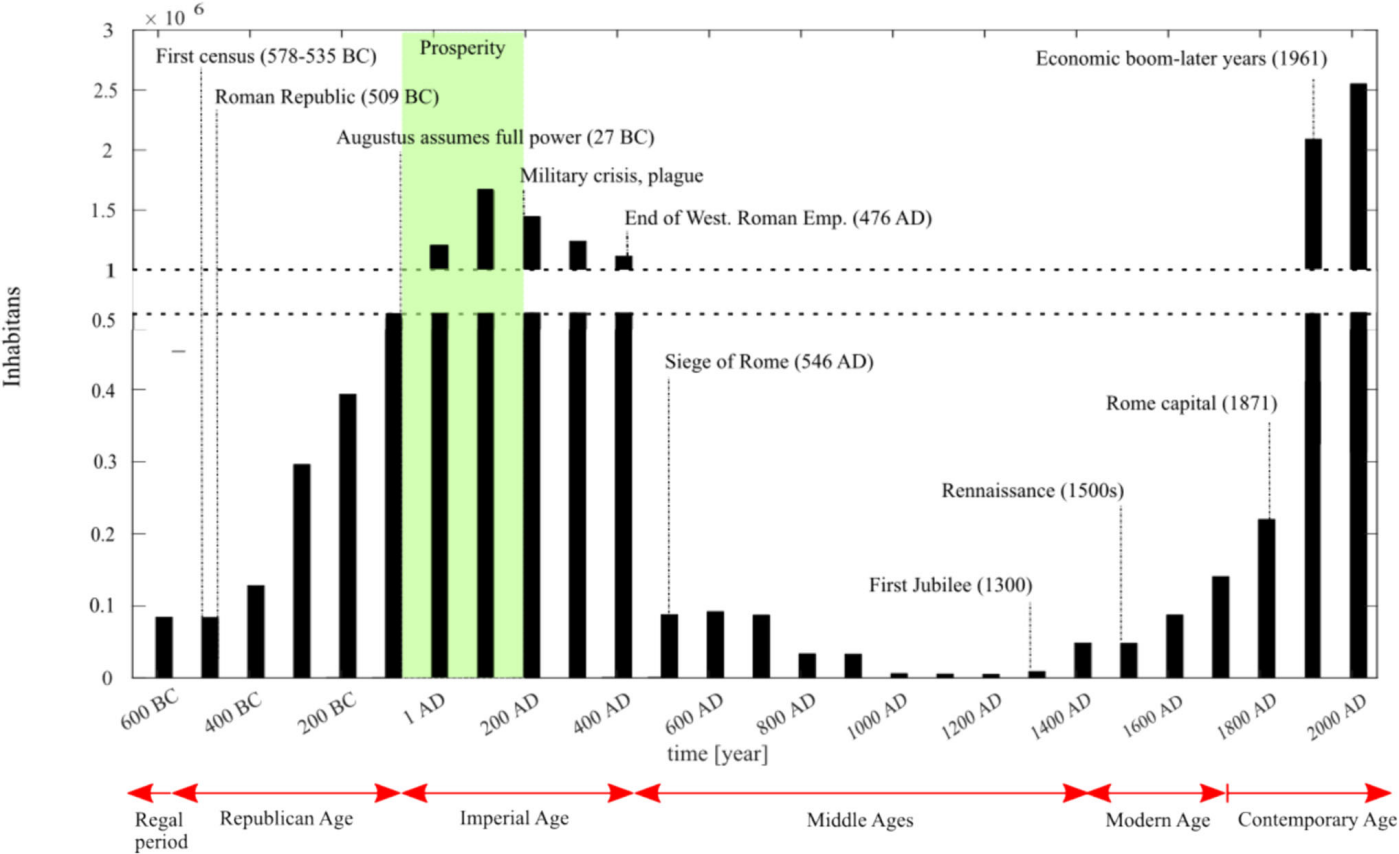
Population estimation in relation to historical events that may have affected it (after Thomas 1989)

Figure 4 shows an estimation of the population from around 600 BC to 2000 AD, and it includes historical events that may have influenced the increase and decrease in the number of people (Thomas 1989). For instance, from 100 BC to around 200 AD, the Roman Empire was at its peak, possibly leading to an increase in population. However, from 180 AD, following Marcus Aurelius's death, a plague began to spread among the population. The plague, combined with the military crisis of the empire, led to a generalized crisis and a significant population decline. AD 476 marked the end of the Western Roman Empire, and Rome entered a "dark" era characterized by ongoing wars, the spread of epidemics, and food production crises, resulting in a reduction in population. Later, in the 1300s, the first Jubilee was celebrated, bringing a wave of pilgrims to Rome. In the 1500s, Rome became the centre of the Italian Renaissance, ushering in a new era of development and prosperity.

The increase and decrease in flood frequency correlate positively with the rise and fall of the population. During the peak of ancient Rome, a large population lived in the city, leading to more potential losses and casualties during flood events. Conversely, the low population period overlaps with the absence of recorded floods. Therefore, the variation in flood frequency may be explained by the small number of people who witnessed, recorded, and, more importantly, were affected by flood events.

Many scholars have also speculated on changes in climate as the main cause of changes in the frequency of floods (Vandenberghe and Maddy 2001). Moreover, anthropogenic changes to the Tiber drainage basin could have been responsible for the change in the pattern of the number of floods. Among the other factors, deforestation may have caused more frequent and severe floods (Delano Smith 1979). The city of Rome required a huge amount of wood for several purposes, such as the construction of buildings, the production of objects, and charcoal production (Hughes and Thirgood 1982). Romans were not aware of the ecological costs of clear-cutting forests and offered incentives to those who transformed woods into farmlands (Meiggs 1982).

## River management dynamics: Adaptation and dominance

In this section, we explore how Roman society responded and adapted to flooding in various ways over time. Many actions and decisions across the centuries suggest the concept of managing flood risk through dual challenges: adaptation, such as elevating the actual ground level, and dominance, exemplified by the construction of key infrastructures.

### Early flood mitigation: Structural and non-structural measures (7th Century BC–4th Century AD)

**Fig. 5:**
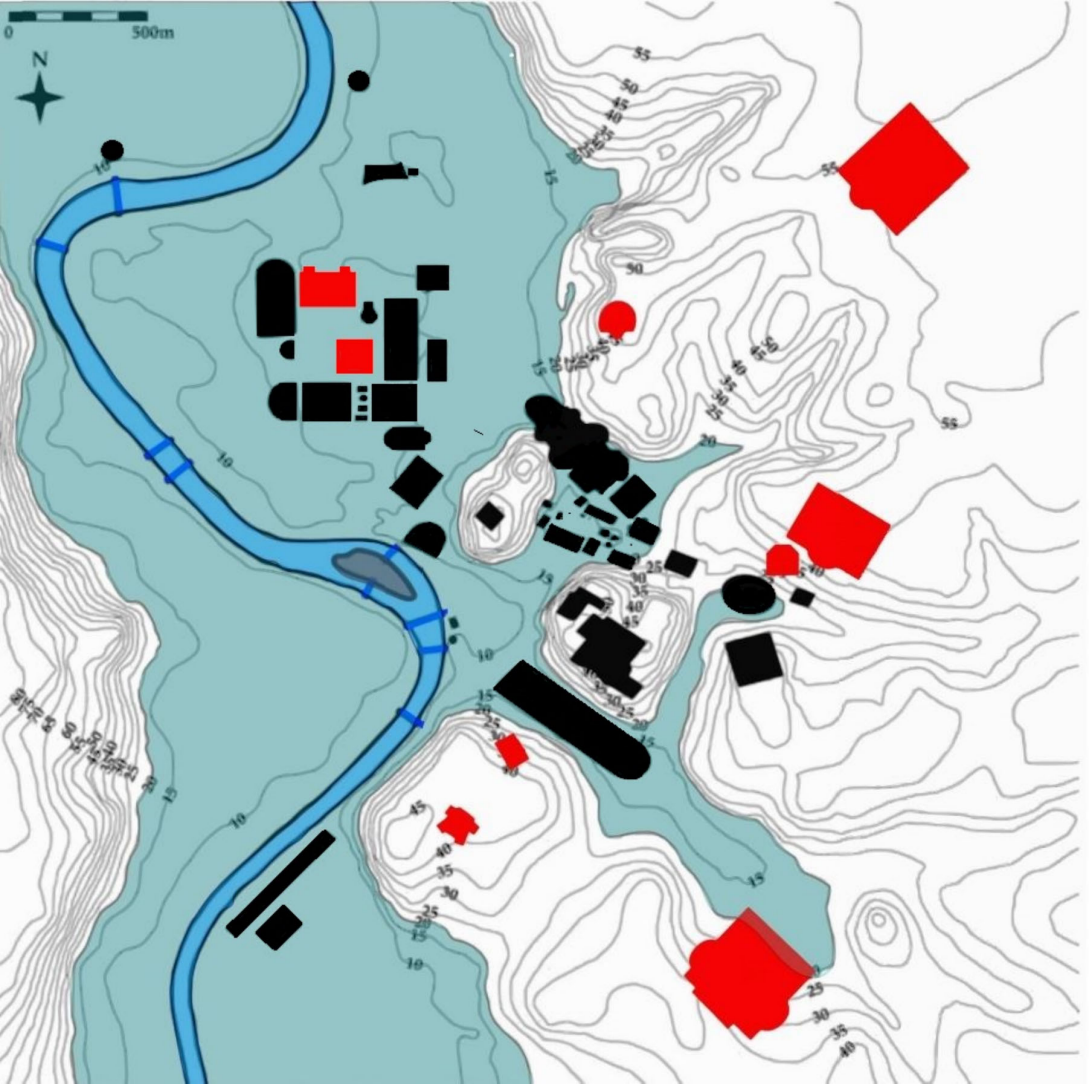
Map showing political, commercial, and entertainment centres of Rome plotted in black, with major bath complexes marked in red plotted, overlaid a 20 m a.s.l. flood level. Baths in black are early construction, while red ones are later constructions, after Aldrete (2007)

In ancient times, Rome faced the challenge of its location on a floodplain, resulting in various parts of the city being vulnerable to flooding, especially during rainy seasons, including the Curia, where the Senate gathered, i.e. the Roman Forum (Ammerman 1990). The first method of taming the river led to the construction of a system of drainage and sewers standing as one of the era's most remarkable engineering projects of the world: the Cloaca Maxima, spanning a considerable length of 1600 m (Mocchegiani Carpano 1984). The later Imperial fora were constructed on slightly higher ground; however, they were not immune to major floods, as shown in Fig. 5. The commercial centre of the city was chosen for easy access to the Tiber, a primary means of transportation. Unfortunately, this condition meant that high-value perishable supplies were stored in flood-prone areas. Not only markets and warehouses, but also Rome's major political, commercial, and entertainment centres were concentrated in areas prone to flooding (Fig. 5).

One of the first attempts of adaptation to make an area less prone to flooding is to raise the actual ground level. The process initiated between the late 7th and early 6th centuries BC, Fig. 3, and continued in the second half of the sixteenth century during the reconstructions made by the Popes, Paul III, and Sixtus V (Dudley 1970; Ammerman 1990; Rubin 2003). Another form of adaptation consisted in building bath complexes on high-elevation sites from the end of the first century A.D. It can be argued that the place of construction of these baths can be seen as a response to population growth, providing essential facilities to newly developed areas. However, Aldrete (2007) suggests that the location is as close to the city as possible while avoiding flood-prone areas, Fig. 5. The same rationale applies to aqueducts: the explanation for their location on the crests of the elevations is twofold. Pipes and conduits would be seriously damaged or polluted by floods; however, their location also provided hydraulic head for water supply in an open-flow system. Regarding the distribution of houses, the private homes (domus), characteristic of the wealthier social class, and apartment blocks (insulae), inhabited by the poorer social classes, were intermingled (Stambaugh 1988; Purcell 1994). The highest concentration of housing coincided with areas with political purposes located near the Tiber River, in the floodplain. This suggests that Roman residential areas were frequently flooded, however, a high percentage of wealthy people resided on hilly areas (Witherstine 1926; Richardson 1992; Reynolds 1996). Even though there are no primary sources reporting that living atop a hill provided protection and benefits in case of flooding, it is not possible to exclude that those locations were chosen also because of the flood-prone nature of the valleys.

Regarding the structural flood control strategies planned in the first century BC, Julius Caesar, one of the most influent ancient Roman generals and statesmen, and a dictator from 49 BC, explored innovative hydraulic solutions to address flooding challenges, including draining the Pomptine marshes in the south of Rome carving a canal through an isthmus to create a secure harbour near Ostia (Suet. Jul. 44). Caesar also suggested to create a new course for the river just upstream from the city centre near the Milvian Bridge, directing it through the Vatican hills via an artificial channel before reconnecting to its original path south of the city (Att. 13.33.4; Favro 1996). His plan was twofold as it aimed at gaining area for enlarging the city and making it safe from flooding; nevertheless, the plan remained unrealized.

Later in time, the emperor Augustus (27 BC14 AD) recognized the importance non-structural flood mitigation measures. Augustus undertook dredging, obstacle removal, and bank clearance to enhance flow capacity and appointed a man to oversight the channel, i.e. the *curator alvei Tiberis* (Suet., Aug., 37; Le Gall 1953a). Following a devastating flood in 15 AD, a five-person committee was established by Tiberius (Dio, 57.14.7–8, Tacitus, Ann. 1.76) and under Trajan (from 98 AD) the office’s duties were extended to the sewers of the city (Sirks 1991; Robinson 1992; Lagunes 2004)*.* A further dredging was performed by Aurelian in the third century AD (SHA, Aurel. 47.2–3), Fig. 3; however, he believed that the intervention would provide a permanent benefit to the river flow capacity, without accounting for the continue necessity of maintain clear the channel, especially since the population continued damping materials into it.

A further structural attempt to alleviate floods in Rome was undertaken by Claudius (41–54 AD). He ordered the construction of a canal connecting the Tiber to a harbour in Ostia (Suet. Claudius. 20). During the second century AD, the Tiber's course underwent changes influencing flood control measures, including Trajan's canal projects targeting excess water release (Le Gall 1953b).

### From the middle ages to an era of reforms and engineering (4th–18th Century)

The Middle Ages are marked by scarce surviving documentation. At the end of the sixth century, Rome was hit by heavy floods, but information gaps arise due to lost medieval documents caused by frequent fires.

The Romans influenced flood events both indirectly, through factors such as inadequate riverbed maintenance, and directly, as their buildings reduced the river’s flow capacity. For instance, the foundations of a circular tower completely closed the two minor arches of the Sant’Angelo bridge that acted as a dam in case of flood causing the inundation of the city centre (Lanciani 1897; Marsico 2018).

In the mid-1500s, during the Renaissance reconstruction of Rome, the Tiber River congestion increased significantly as both a consistent number of boats shipping construction materials and water mills disrupted the flow (Bersani and Bencivenga 2001). Additionally, the riverbed became a dumping site, further impeding flow (Pensuti 1925).

Floods occurred in 1530 and 1557 underscored the necessity of non-structural mitigation measures. The urban expansion far away from the river promoted by the Popes Gregorio XIII and Sisto V decreased the vulnerability of the floodplain, thus mitigating the effects of future flooding (Di Martino and Belati 1980). Furthermore, the channel maintenance was restored.

Among the events that reduced the occurrence of floods, we can mention a flood that cut a meander near the river mouth in 1562 (Guglielmotti 1893; Tomassetti 1897). Later, during the 1598 flood, which had an estimated 1000-year return period, two pylons of the Senatorial bridge collapsed on the left bank, thereby alleviating the riverbed from a major obstruction (Di Martino and Belati 1980). Furthermore, in the late fifteenth century, the removal of floating mills, the riverbed cleaning, and the demolition of the circular tower at Sant’Angelo bridge in 1623 improved the flow capacity (Evans 2002). In the early seventeenth century, Popes Gregorio XV and Urbano VIII restructured the sewerage system, enhancing Rome’s flood resilience (Bersani and Bencivenga 2001).

### Modern flood defences and reservoirs (19th Century)

In 1870 an exceptional flood event caused an extensive inundation of Rome and prompted the construction of contemporary flood walls. These walls, which towered 10 to 11 m high, were erected on both sides of the river.

The plan included the construction of flood walls on both river sides in the urban section, following the removal of obstructions. The left bank was embanked up to the area downstream from Marconi Bridge. Two parallel sewers were constructed along the riverbanks, with the right sewer connected to the left via a siphon, extending the first sewer to a point where backflow during floods is no longer a concern. The construction concluded in 1926 (D’Onofrio 1980), despite not being fully completed, its presence prevented extensive damage during the 1915 flood (Bersani and Bencivenga 2001).

Demographic growth following Italy's Unification in 1870, coupled with wall construction, fuelled urban expansion, even in flood-prone zones (Ferrarotti 2003). Consequently, the construction of flood walls also leads to a significant increase in exposure to flood. In facts, Rome stands out as a unique case in the history of urban planning in Italy, with its population growing from 226,000 in 1871 to over 2 million during the Italian economic boom that occurred from the 1950s to the early 1960s (Fig. 4, ISTAT 2021).

Subsequently, following the 1937 flood, an artificial canal was constructed to divert a meandering section of the Tiber River located between the city and its mouth. From the 1950s, the construction of large multi-purpose reservoirs upstream from Rome regulates the flow, but also reduces the sediment load (Calenda and Mancini 1999), that, in turn, leads to the deepening of the riverbed, challenging the bridges’ piers (Biscarini et al. 2021).

## Flood risk perception in modern times

To assess Roman attitudes toward flooding in ancient times, in the previous sections we have explored how the flood-prone nature of the area influenced the way the city developed, and we analysed the adoption of structural and non-structural mitigation measures over time. Nevertheless, it is not possible to speculate about flood risk perception and preparedness in ancient times. On the contrary, in recent years there has been a rising interest in investigating the topic in Rome (De Dominicis et al. 2015).

According to Slovic (1987), risk perception is based on individual judgment in a context that may be characterized by uncertain information. Several aspects impact risk perception, such as frequency, seriousness, and direct/indirect experience of risk events (Bronfman et al. 2020).

The connection between perception and preparedness remains unclear, yet it is acknowledged as an initial stride towards enhancing flood response (Scolobig et al. 2012).

Mysiak et al. (2013) examined risk perception, relative concern, and experiential factors among residents of flood-prone areas in Rome. They investigated whether individuals displayed an elevated level of awareness in response to increased hazard and whether this heightened awareness resulted in more effective risk mitigation strategies. Heightened risk perception and concerns were evident in areas characterized by greater hazard levels. Nonetheless, measures of behavioural intention did not exhibit significant variance.

To untangle the link between awareness and preparedness, De Dominicis et al. (2015) investigated the relationship between place attachment and risk perception in Rome. Place attachment is defined as the global feelings, bonds, and behaviours that people develop in relation to their social and physical environment and may determine specific place-related behaviours, in turn associated with place-specific biases (e.g. Twigger-Ross and Uzzell 1996; Brown and Perkins 1992). De Dominicis et al. (2015) found that people with high neighbourhood attachment show high flood risk perception and related flood concern but only when they live in low-risk areas. Conversely, in high-risk areas, place attachment may lead to a false sense of safety that induces people to be not fully aware of the actual risk.

To assess the role of the flood walls in shaping flood risk perception in Rome, Ciullo et al. (2017) analysed historical data and developed a socio-hydrological model representing the changing flood risk from the 1800s to contemporary Rome. The model aims at understanding how the impacts and perceptions of floods have influenced local demography, while policies and measures of flood risk mitigation have, in turn, affected the frequency of floods. As soon as the community in Rome experiences a flood event, it builds memory of the flooding, which is a crucial resource when making decisions on how to manage flood risk. After the occurrence of the event, the memory decays over time (Hanak 2011). Since flood events may be experienced by Rome’s inhabitants only when the levees are overtopped by high waters, the construction of flood walls hinders the assessment of the collective memory of recent flood events (Di Baldassarre et al. 2017b). It was found that prolonged phases of relative “calm” can diminish the flood memory that local inhabitants have cultivated following the experience of flood events (Ullberg 2018).

The concept of flood memory has demonstrated a notable impact on communities’ resilience. Insufficient levels of flood memory could elevate vulnerability and potentially facilitate inadequate responses to hydrological extremes (Garde-Hansen et al. 2017). These findings highlight the importance of enhancing flood risk communication in Rome as a pivotal strategy for raising awareness and bolstering preparedness.

## Discussion

Based on primary sources, Rome seems to have been a resilient city that displayed adaptability in response to extreme events. The vulnerability of ancient Rome could have been minimal due to five characteristics of the city itself: topography, construction methods, residential housing patterns, secure food storage, and a continuous water supply that suggest a high level of flood risk awareness. However, Romans did not construct structural protection measures, despite having the financial and technical capacity to set in place a comprehensive levee system for the city's protection. Among the diverse reasons, it is worth mentioning that the Tiber River was considered on par with divinity, and some changes could not be performed because of it. Therefore, Roman engineers had to face the risk of divinities' disfavour while pursuing their duties (Holland 1961). Centuries later, the construction of the flood walls not only modified flood risk in Rome but also altered the city itself by transforming its relationship with the Tiber. If in previous centuries the Tiber was at the centre of the city's economic and social life, the construction of the walls placed a barrier between the buildings and the river waters. On the other hand, however, it was precisely the construction of this barrier that allowed urban development in areas that had previously been sparsely populated or even used as a buffer for the flooding waters. Specifically, Milvian Bridge is the first river cross section upstream from the flood walls that exhibits critical issues before the river enters the stretch flowing through Rome. From Milvian Bridge up to Marconi Bridge, the urban stretch is surrounded by the flood walls. Conversely, in the stretch flowing through the city centre, the Tiber Island is an area prone to high flood risk that is not protected by levees and whereby the presence of a hospital facility increases the vulnerability of the site. A further site characterised by high flood risk is at the mouth of the Tiber River, posing threats to nearby communities and infrastructures (Protezione Civile 2024).

The current increase in the frequency and intensity of extreme events linked to global warming, along with the increase in exposure and vulnerability due to population growth and aging infrastructures (Aghakouchak et al. 2020; Fiori et al. 2023; Moccia et al. 2024), emphasizes the need for policies and interventions that can mitigate residual risk.

The presence of flood mitigation structures triggered behavioural feedback leading to increased development in hazardous areas now protected. Hard-adaptive measures, such as flood walls, may become maladaptive as they can unintentionally heighten vulnerability and exposure, a phenomenon known as “levee effect” (Fusinato et al. 2024). Conversely, heightened hazard awareness, which is a form of soft adaptation, can decrease vulnerability. Disregarding behavioural feedback in hazard assessment has the potential to fail in detecting maladaptive actions (Logan et al. 2018). Indeed, the literature review shows that in Rome, the presence of flood walls may alter flood risk perception, and a longer interarrival time between flood events may fade the memory of locals (Di Baldassarre et al. 2017b). Therefore, maintaining a strong memory of past flood experiences is a key factor in boosting preparedness (e.g. Mysiak et al. 2013; De Dominicis et al. 2015; Ciullo et al. 2017).

Success stories of flood risk mitigation have in common integrated management, leading to the implementation of structural and non-structural measures, including improved early warning systems and participative risk awareness programs (Kreibich et al. 2022). Nowadays, for risk assessment and management, reference is made to the EU Directive 2007/60. Within this framework, the Civil Protection has issued a plan for risk mitigation in the entire territory of Rome, combining structural (i.e. flood walls) and non-structural measures (e.g. early warning systems, educational programme for the population). However, it is worth noting that flood risk mitigation strategies were issued long before the EU Directive and from 1999, the Basin Authority of Tiber River has carried out specific studies and planned interventions, coupled with urban planning, to ensure that new developments account for flood risk. Early warning systems monitor weather patterns and river levels in real time, providing timely alerts to residents and authorities, allowing for prompt evacuations and preparatory actions. Moreover, education programs in schools increase population awareness, fostering a culture of preparedness among the younger generation. Public awareness campaigns, workshops, and participatory planning processes ensure residents are informed about risks and protective measures. This participatory approach improves the effectiveness of risk mitigation measures and strengthens community trust and cooperation.

## Conclusions

The understanding of coupled human-flood systems can inform flood management decisions and provide insights into potential future phenomena, such as the adaptation effect or a shift from flood risk to environmental concerns and support integrated flood risk management. A dynamic approach involving human-flood systems can facilitate the examination of potential future scenarios resulting from the interactions among the system's various components. Neglecting the future dynamics of urban development and the temporal evolution of risk can lead to erroneous conclusions regarding adaptation strategies. Consequently, incorporating these processes becomes an imperative consideration for understanding how hazards impact communities.

This paper explores the intricate, evolving relationship between the Tiber River and the city of Rome over the centuries, revealing how Rome's resilience and adaptability to flood risk have been shaped by both adaptive and dominant strategies. Initially, the Romans addressed flood risk through adaptation—enhancing river cross-section capacity and elevating wooden house floors—while also asserting dominance by constructing major infrastructures like diversions, canals, and, eventually, flood walls.

This paper demonstrates that the construction of flood walls in modern times marked a turning point, ushering in a new era of urban development but also fundamentally altering the city's relationship with the Tiber. While these walls provided protection, they may have inadvertently weakened local flood risk awareness, potentially leading to inadequate responses during possible disasters. The “levee effect” highlights that structural solutions alone are insufficient; effective risk mitigation requires a combination of structural and non-structural measures.

In contemporary Rome, the Civil Protection has recognized the importance of integrating these approaches. They have enhanced non-structural measures, such as refining early warning systems that issue timely alerts to authorities and citizens, and ensuring riverbanks are inaccessible during floods. Education programs in schools are also crucial, fostering a culture of preparedness among younger generations through targeted awareness initiatives.

Ultimately, this study underscores that successful flood risk mitigation in Rome hinges on a balanced strategy that combines robust infrastructure with ongoing public education and engagement to maintain high levels of risk awareness and preparedness.
