# Publisher Correction: The interplay between the urban development of Rome (Italy) and the Tiber River floods: A review of two millennia of socio-hydrological history

**DOI:** 10.1007/s13280-024-02095-4

**Published:** 2024-11-21

**Authors:** Elena Ridolfi, Mara Lucantonio, Giuliano Di Baldassarre, Benedetta Moccia, Francesco Napolitano, Fabio Russo

**Affiliations:** 1 Dipartimento di Ingegneria Civile, Edile e Ambientale, Università Degli Studi di Roma La Sapienza, 00184 Rome, Italy; 2 Department of Earth Sciences, Uppsala University, Uppsala, Sweden; 3 Centre of Natural Hazards and Disaster Science, Uppsala, Sweden

**Correction to: Ambio** 10.1007/s13280-024-02078-5

In the original published article, the author name “Fabio Russo” was incorrectly written as “Francesco Russo”. Also the caption of figure 2 is updated from “Extent of the Roman Walls over the centuries” to “Extent of the Roman Walls over the centuries (after Canina, 2015)”.

The original article has been corrected.

